# Efficient End-to-End Convolutional Architecture for Point-of-Gaze Estimation

**DOI:** 10.3390/jimaging10090237

**Published:** 2024-09-23

**Authors:** Casian Miron, George Ciubotariu, Alexandru Păsărică, Radu Timofte

**Affiliations:** 1 Faculty of Automatic Control and Computer Engineering, “Gh. Asachi” Technical University of Iaşi, 700050 Iaşi, Romania; timofte.radu@gmail.com; 2 MCC RESOURCES S.R.L., 707406 Iaşi, Romania; 3 Computer Vision Lab, CAIDAS & IFI, “University of Würzburg”, 97070 Würzburg, Germany; 4 Faculty of Electronics, Telecommunications and Information Technologies, “Gh. Asachi” Technical University of Iaşi, 700050 Iaşi, Romania

**Keywords:** computer vision, convolutional neural networks, point of gaze, deep learning

## Abstract

Point-of-gaze estimation is part of a larger set of tasks aimed at improving user experience, providing business insights, or facilitating interactions with different devices. There has been a growing interest in this task, particularly due to the need for upgrades in e-meeting platforms during the pandemic when on-site activities were no longer possible for educational institutions, corporations, and other organizations. Current research advancements are focusing on more complex methodologies for data collection and task implementation, creating a gap that we intend to address with our contributions. Thus, we introduce a methodology for data acquisition that shows promise due to its nonrestrictive and straightforward nature, notably increasing the yield of collected data without compromising diversity or quality. Additionally, we present a novel and efficient convolutional neural network specifically tailored for calibration-free point-of-gaze estimation that outperforms current state-of-the-art methods on the MPIIFaceGaze dataset by a substantial margin, and sets a strong baseline on our own data.

## 1. Introduction

In recent years, the proliferation of e-meeting platforms has underscored the critical need to enhance user experience in these settings. Visual attention analysis has surged in popularity, demanding the development of more robust real-time machine learning models and the acquisition of gaze-point estimation data from basic consumer-grade RGB sensors. While similar applications like gaze-based human–computer interaction and user-state awareness have been extensively explored, point-of-gaze (PoG) estimation remains a persistent and crucial issue in both academic and industrial domains. Furthermore, PoG estimation exhibits significant promise in medical applications, especially for patients with paralysis. By facilitating gaze-based control of a virtual keyboard, these patients can manipulate the cursor across the screen and form words, providing a fundamental mode of communication. This technology offers an essential means of interaction and expression for individuals who may otherwise lack the ability to communicate verbally or physically.

On the one hand, up to this date, lots of applications employ infrared (IR) pupil tracking [1] for acquiring ground truth labels. Moreover, other techniques include intrusive wearable gear, such as headsets [2], for closely monitoring the subjects. Unfortunately, they require specialized hardware components that are not cost-effective or competitive with the latest advancements in RGB sensors in consumer-grade cameras. Furthermore, the procedures for gathering these datasets and the necessary training the subjects must go through are cumbersome and they hinder fast data acquisition.

On the other hand, numerous approaches choose to opt for a calibration-based method [2,3,4], so that they can achieve competitive results. However, this may result in suboptimal use of computational resources, due to the required deep convolutional neural network (CNN) architectures for processing all the information from a mixture of inputs from multiple sensors, using known calibration parameters.

Stemming from these aforementioned observations based on recent state-of-the-art (SotA) works, our contribution is twofold: (1) we propose a streamlined, time-effective, and lightweight data gathering methodology for accurate ground truth annotations, and provide the scientific community with a proof-of-concept dataset for PoG estimation consisting in more than 20,000 samples of multiple unrestricted subjects captured in very diverse real-world settings; and (2) we carefully analyze the problem and come up with a calibration-free end-to-end CNN-based efficient method for PoG estimation that can outperform all the compared methods from the literature and achieve SotA performance on the MPIIFaceGaze dataset [5], and offer a solid baseline on our novel dataset. Despite the remarkable results, our architecture is a remarkably simple feed-forward network that relies on convolutional blocks, rectified linear unit (ReLU) activations, and batch normalization (BN) or dropout techniques.

The structure of the rest of this paper is presented in the following lines. Firstly, Section 2 offers an overview of the several papers we compare our work to. Then, in Section 3 our methodology for the model design is described. Afterwards, Section 4 prepares the ground for the dataset acquisition and quantitative and qualitative description. Next, Section 5 presents our model’s results on the MPIIFaceGaze dataset [5] as well as an ablative study on our own data; this is followed by Section 6, which presents an overview of our work compared to the literature and presents possible future improvements. Lastly, Section 7 sums up our contributions and expands the scope of our research.

## 2. Related Work

One notable dataset in eye-tracking research is EVE [1], which involves a complex methodology for recording ground truth labels. Its setup incorporates a machine vision camera, a near-IR eye tracker, and three webcams, which all require thorough synchronization with the screen. Despite the intricacy of this arrangement, it operates under static lighting conditions within a controlled laboratory environment, and the subjects are placed about 40–80 cm from the cameras. Lastly, their GazeRefineNet model approach does not use the plain raw image, but the segmentation of the two eyes to predict the PoG, which complicates the ground truth (therefore requiring additional annotations) or requires specialized models for estimating these segmentation masks.

Another approach that uses the aforementioned dataset is end-to-end frame-to-gaze estimation, namely, EFE [6], which predicts the gaze vector (3D origin and 3D direction). Although it uses the entire image for making predictions, the resolution it requires for achieving competitive results is too large to be able to perform in real time.

Moving on to another dataset, MPIIGaze is used to talk about [4], and MPIIFaceGaze [5] to designate the annotated subset of MPIIGaze. It is a complex dataset that required an acquisition duration of over 45 days for 15 participants in daily-life conditions. The collection procedure is complex and requires a lot of effort from the user side. Furthermore, each RGB webcam’s intrinsic parameters are required for thorough calibration. Additionally, the complex pipeline for face and landmark detection makes their processing pipeline rather complex and error-prone. Their eye image segmentation multimodal CNN model outputs the gaze vector, but it requests many known priors and vast computational power for accurately predicting the essential upstream features.

A method that uses the previous dataset is full-face appearance-based gaze estimation [5]. We had a similar idea for designing the encoder architecture. However, the main difference is that the authors introduce an extra skip connection that reduces their model’s efficiency, on top of the fact that they plug more layers into their architecture. Moreover, they use only segmented face images as input for their model, which requires a camera model with fixed intrinsic parameters.

A different eye-tracking setup is Pupil [2]. It leverages an IR-spectrum sensor mounted onto a headset designed for “dark pupil” detection at 30 Hz. The eye camera image is converted to grayscale. Also, their proposed algorithm detects the pupil’s contour by proposing candidate ellipses. A subject wearing the Pupil Pro eye tracker sits approximately 0.5 m away from a 27-inch computer monitor, ensuring a wider variety of gaze points. The eye-tracker is calibrated using the standard nine-point screen marker-based calibration routine running in full-screen mode.

Lastly, our discussion focuses on the GazeCapture large-scale dataset [7], collected using crowdsourcing. The paper also introduces the iTracker model. Unfortunately, their setup is only designed for iOS devices. Furthermore, it restricts its users by requiring airplane mode during the data acquisition process. Their methodology consists in requiring the user to follow a 2-second procedure for acquiring one image, and more interactions for completing the operation, which yields more accurate results at the expense of being extremely time consuming.

## 3. CNN Methodology

Having enumerated the advancements of previous works, the current state of knowledge in PoG estimation has been presented. Therefore, the specifics of our contribution are put forward, namely, our fully convolutional efficient architecture for solving the aforementioned task.

The proposed architecture is depicted in Table 1. This CNN architecture is designed for tasks involving image processing, particularly gaze estimation or similar tasks that involve image-based inputs. To further clarify our choice of architecture design, a deep dive into each layer block’s role is performed, including their parameters and how they contribute to the network’s overall functionality.

- Block 0 (input image): The journey begins with the input layer, where the network receives an image with dimensions H×W×3, representing its height, width, and RGB color channels, respectively. This raw image is then fed into subsequent layers to extract meaningful features.
- Block 1 (convolution + ReLU + dropout + maxpooling): The first convolutional operations introduce the first block of computations. A convolutional layer with a 3×3 kernel and 64 filters performs feature extraction. Each filter scans through the image, detecting patterns like edges or textures. The rectified linear unit (ReLU) activation function follows, introducing non-linearity by replacing negative values with zeros. This block’s purpose is to capture weakly processed shallow features from the image. Additionally, dropout is applied with a probability of 0.3 during training, randomly setting some activations to zero, hence preventing overfitting. The maxpooling layer reduces the spatial dimensions by half, employing a 2 × 2 kernel with a stride of 2. It retains the most significant features from the previous layer while reducing computational complexity. Consequently, the output shape becomes ((H − 2)/2) × ((W − 2)/2) × 64. Owing to this feature map compression, the model can further focus on the most relevant information.
- Block 2 (convolution + ReLU + dropout + BN): Continuing the pattern, the second block adds another convolutional layer with the same 3 × 3 kernel and 64 filters. The ReLU activation maintains non-linearity, and dropout regularizes the model. This layer further refines the extracted features, capturing more intricate details. Batch normalization (BN) improves training stability and speed by normalizing the input of the previous layer. It reduces internal covariate shift, making the network more robust and accelerating convergence. With 256 parameters, BN maintains consistency in the network’s behavior.
- Blocks 3–4 (repeated blocks): The following blocks belong to a series of repeated blocks, each containing convolution + ReLU + dropout + batch normalization. These blocks deepen the network’s representation capability. While maintaining the size of the feature maps for now, the 3 × 3 convolutional filters with 64 channels capture hierarchical features, while ReLU ensures non-linear transformations. Dropout adds robustness against overfitting, and batch normalization maintains stable gradients, as previously mentioned.
- Blocks 5–11 (more repeated blocks): These blocks are composed of the same base elements as the previous set of blocks. However, the main difference is that the output dimensions gradually decrease as the layers progress. This enables the network to focus on more abstract and higher-level features, thanks to the representation size compression. This leads to the selection of the most discriminative features for enabling the model to perform the best.
- Block 12 (global average pooling + dropout): Global average pooling condenses the spatial information into a single vector by averaging each feature map’s values. This step reduces the output to a fixed size (64), making the network invariant to input size variations and reducing computational complexity. A dropout layer with a probability of 0.3 is applied once more. This further improves the model’s generalization by preventing reliance on specific features.
- Block 13 (dense): After having applied a flatten layer, the final dense (fully connected) layer, with an output size of 2, connects all neurons from the previous block. With 130 parameters, the dense layer processes the highly processed condensed features to make the final prediction.
- Output block: The output layer has no final activation function. It outputs the model’s final prediction, which corresponds to the precise point-of-gaze (PoG) coordinates, on the screen.

**Figure 1:**
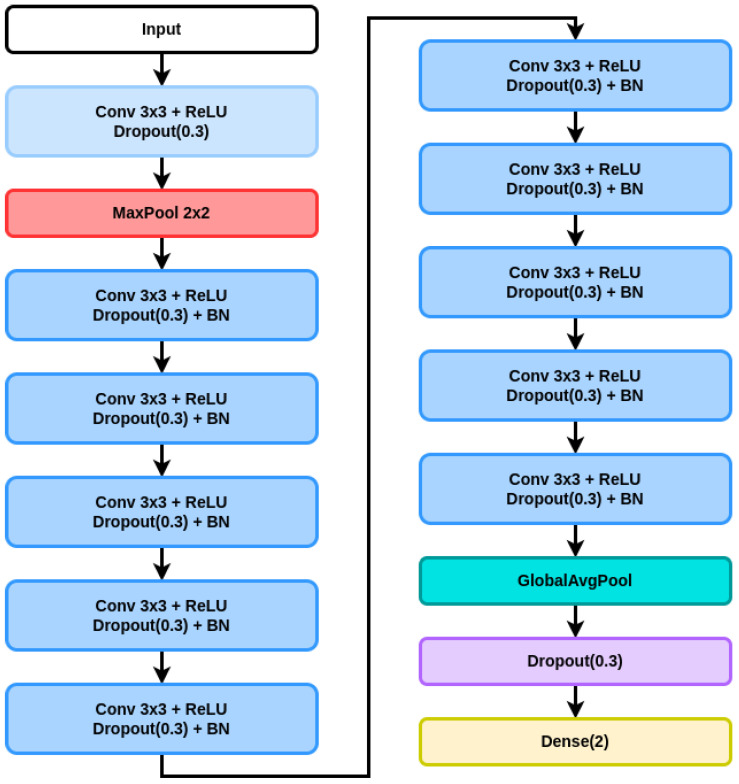
Proposed CNN architecture for PoG estimation.

One important aspect is that the maxpooling layer is graphically represented as a separate computational block in Figure 1, to underline the differences between the first computational block and the next ones. As observed in Table 1 as well, there is the input layer denoted by “Block 0”, then the first computational block is colored in light blue, as it does not include a BN operation, but it includes the maxpooling layer that halves the feature map’s height and width. Next, 10 similar convolutional blocks follow, which are aimed at extracting informative regional dependencies of highly processed features. Lastly, blocks 12 and 13 are depicted as the last three graphic elements of Figure 1, and they are designed to distill all the information into the most condensed representation, so that the model yields the best results.

All in all, this CNN architecture employs a series of convolutional, pooling, dropout, and batch normalization layers to progressively extract intricate features from the input image. With a total of 373,762 parameters, the network is designed for tasks like gaze estimation, where understanding the subtle details of facial features is crucial. Through repeated blocks and pooling layers, the model learns hierarchical representations, culminating in a compact and efficient architecture capable of accurate predictions in tasks requiring image analysis.

Overall, our informed decisions and extended experiments helped us choose our inherently efficient design. This resulted in achieving a real-time-capable model while having the advantage of being more resource-efficient by a large computational margin compared to previous works, thus underlining the importance of our contribution.

## 4. Dataset Benchmarks

This section aims to cover the particularities of several datasets from the literature, and converge towards understanding our methodology for gathering our data and organizing it. Lastly, we mention how we used it for training our model and what data pre-processing was performed to improve the results.

### 4.1. PoG Estimation Datasets

Firstly, this subsection sets out our data acquisition procedure, together with the dataset’s specific quantitative features. Then, other relevant datasets from the literature are briefly presented, and then, compared to our work.

#### 4.1.1. Data Collection

Until recently, multiple works have used cumbersome procedures that were designed to be either user-friendly or to ensure the quality of the gathered data, but not both at the same time. Notably, several works from the literature make use of intrusive eye-tracking devices that must be worn by the subjects [2], or they employ specialized infrared (stereo) cameras for pupil detection and tracking [1]. This unnecessarily increases the complexity of the data collection procedure for PoG estimation samples. We aim to get rid of the wearable gadgets, stereo trackers, and complex client apps that result in suboptimal acquisition procedures (too many checks, need for thorough calibration, too many error sources) [7]. Therefore, not only do we design a lightweight and effective pipeline that does not restrict the user in any way, and does not reduce the diversity of random in-the-wild conditions, but also it does not require calibration of any kind, and it is exceptionally fast and user-friendly. Instead of instructing the user to comply with lots of constraints and spend a lot of their time on complicated procedures, we provide them with a desktop client that lets them click on random points on the screen while focusing on them.

**Figure 2:**
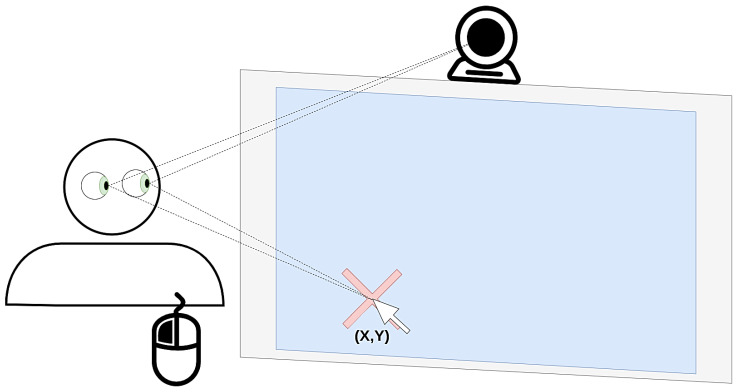
Data acquisition setup explained: The subject stares at their mouse pointer, points and clicks on their screen, the coordinates of the point are stored in the database, and the webcam captures a photo.

To develop a dataset for estimating the gaze point on the device’s monitor, we engineered a software client that leverages the webcam to capture subjects’ images while they gaze at the display. A graphical representation is provided in Figure 2. In the experiment, each subject was instructed to closely follow the tip of the cursor on the screen. When they pressed the left mouse button to indicate their selection, an image was automatically captured using the webcam. The x- and y-coordinates of the mouse cursor were saved alongside the captured image, providing crucial information regarding the subject’s gaze point within the screen frame. This interactive and straightforward method facilitates the collection of necessary data for training and evaluating gaze estimation models, contributing to the advancement of human–computer interaction technologies.

#### 4.1.2. Dataset Characteristics

**Figure 3:**
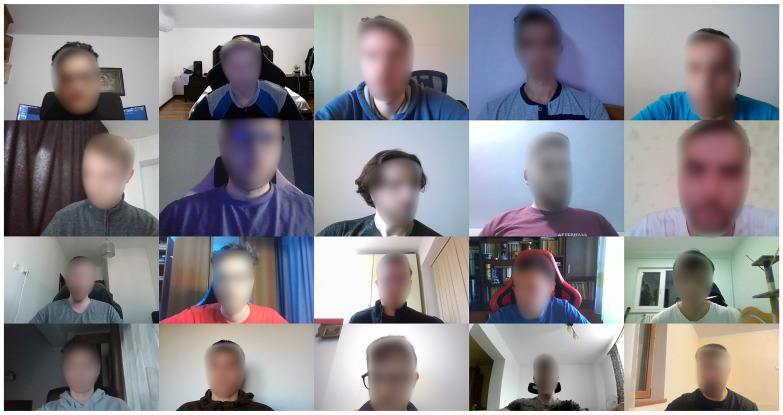
Sample images from our novel dataset: candidates’ faces have been blurred for display in the main paper; however, the dataset contains the original versions. The different resolutions and aspect ratios are noticeable. Additionally, one can observe the complex and diverse real-world conditions in which the images were recorded.

Since we designed a streamlined data acquisition pipeline, which ensures the ground truth annotations’ quality, our chosen subjects successfully minimized gaze coordinate error and collected a significant amount of data in a relatively short time. As a result, in this study, we amassed a dataset comprising 20,221 images from 33 individuals, comprising 7 females and 26 males, aged between 20 and 28 years, with 10 of them wearing glasses. Each individual contributed approximately 600 images. We obtained GDPR-compliant consent from all the participants involved, with them therefore agreeing to be part of the database and allowing their faces to appear, if necessary, in a blurred form in scientific publications. Regarding ethical approval, the study did not require formal approval from an ethics committee. However, all participants signed informed consent forms that detailed the nature of the experiment, their involvement, and the potential use of their images in research outputs, in full compliance with GDPR requirements and ethical standards. The dataset was partitioned into three subsets: one for training, one for validation, and one for testing. The validation and testing sets each comprised 1650 images, with 50 images per individual, while the remaining 16,921 images were allocated for training. The distance between the subjects and the monitor ranged from 20 cm to 80 cm. Source screen resolutions spanned between 1366 × 768 and 2880 × 1800 pixels, with diagonal screen sizes varying from 14 inches to 31.5 inches. Due to some errors in image scaling, these height and width values may differ by a few pixels in our dataset. Each subject utilized their own device and webcam, thus substantially increasing the diversity of the collected data. Images were acquired from diverse angles and under varying lighting conditions and backgrounds. Depending on the webcam used, image resolutions ranged from 640×480 to 1920×1080 pixels. Samples of the images captured during the data collection process can be found in Figure 3. Participants were responsible for managing their breaks and photo sessions. They were free to take breaks as needed and to control the pace of their data collection, ensuring they could perform at their best without intervention from the experimenters. This comprehensive dataset facilitates the development and evaluation of gaze estimation models across a wide range of real-world conditions, encompassing diverse hardware configurations and environmental factors.

Considering the presented description of our dataset, Table 2 provides us with a further detailed report regarding the involved subjects in our study and the particularities of each recording session. Hence, the dataset information encompasses height and width in pixels and millimeters; mentions the human-to-screen distance in cm, presents the webcam resolution, and finally, shows us how many pictures have been taken of every subject. The last line displays the total count of samples collected so far. The dataset is restricted due to privacy and ethical considerations. Researchers interested in accessing the data should contact the corresponding author for further information and potential access. Any data sharing will be subject to approval and may require adherence to specific ethical guidelines and confidentiality agreements.

#### 4.1.3. MPIIFaceGaze Dataset

As mentioned in Section 2, the MPIIGaze dataset [8] contains 213,659 images collected from 15 participants in daily-life conditions, over more than three months. The number of images collected by each participant varied from 1498 to 34,745. However, the useful subset that is considered in our experiments is the one dubbed MPIIFaceGaze [5], which consists of 37,667 annotated images for our task of choice. We have chosen this dataset for comparing our method to other works, based on the authors’ claim that it is significantly more variable than existing ones concerning appearance and illumination.

Although the dataset is designed for more complex 2D and 3D tasks, only the on-screen PoG estimation is considered. Nonetheless, the data are split into “original” and “normalized” for all the 15 participants, and organized by day. The “original” folders are the cropped eye rectangle images with the detection results based on the face detector [9] and facial landmark detector [10]. The “normalized” folder, containing a 6-point-based face model, can be found in the annotations used for the proposed model. For each day, there is an “annotation.txt” file that has information such as detected eye landmarks, on-screen gaze target, gaze vector, and 6-point-based 3D face model, as in [11], or estimated eye centers.

### 4.2. Implementation Details

PoG prediction using CNNs usually requires a lengthy network training process to obtain a low Euclidean distance. We propose an efficient PoG estimation method that requires a short time interval for training and offers Euclidean distance values that represent a reliable PoG prediction. The PoG coordinates are normalized in the range of [0,1]. To normalize the coordinates (x,y) of a point on the screen to a range [0,1] based on the screen resolution $\left(x_{\mathrm{width}},y_{\mathrm{height}}\right)$, one can use Equations (1) and (2). For MPIIFaceGaze, the training is terminated after 30 epochs. The network’s input is a webcam frame resized to 128 × 128 pixels from the initial resolution in the original dataset. Because the images have a lot of background information and the dataset is theoretically small for an end-to-end approach, we use the model trained on MPIIFaceGaze as a starting point (pre-trained model) and we fine-tune it for 15 epochs on our own dataset to decrease the value of the PoG estimation mean square error. Additionally, the CutOut [12] augmentations are used during training, with a probability of 50%.

(1) $$x_{\mathrm{normalized}}=\frac{x}{x_{\mathrm{width}}}$$

(2) $$y_{\mathrm{normalized}}=\frac{y}{y_{\mathrm{height}}}$$

where:

- x,y are the coordinates of the point on the screen in pixels;
- $x_{\mathrm{width}}$ is the width of the screen in pixels;
- $y_{\mathrm{height}}$ is the height of the screen in pixels;
- $x_{\mathrm{normalized}}$ is the normalized x-coordinate in the range [0, 1];
- $y_{\mathrm{normalized}}$ is the normalized y-coordinate in the range [0, 1].

## 5. Evaluation

In this section, the focus lies on providing reliable and fair quantitative and qualitative results on well-known benchmarks, as well as on our novel dataset. To evaluate the performance of our efficient CNN solution, we adhere to the point-of-gaze literature and employ one indicator (i.e., Euclidean distance), and also report the number of parameters for each CNN-based solution. The Euclidean distance is defined as follows:

(3) $$d=\sqrt{{\left(x_{2}-x_{1}\right)}^{2}+{\left(y_{2}-y_{1}\right)}^{2}}$$

where:

- $x_{1},y_{1}$ are the coordinates of the predicted point;
- $x_{2},y_{2}$ are the coordinates of the real point;
- $\left(x_{2}-x_{1}\right)$ is the difference in the x-coordinates of the two points;
- $\left(y_{2}-y_{1}\right)$ is the difference in the y-coordinates of the two points;
- ${\left(x_{2}-x_{1}\right)}^{2}+{\left(y_{2}-y_{1}\right)}^{2}$ is the sum of the squares of the differences in the x- and y-coordinates;
- $\sqrt{{\left(x_{2}-x_{1}\right)}^{2}+{\left(y_{2}-y_{1}\right)}^{2}}$ is the Euclidean distance.

### 5.1. Comparative Results on MPIIFaceGaze Dataset

In order to provide a fair comparison with existing SotA methods from the literature, the evaluation methodology from [6] has been adopted. Namely, a 15-fold cross-validation is performed, so that the chances of having an overfit checkpoint decrease, and consequently, our model is appropriately fitted to the data.

Thus, Table 3 presents the works and the proposed architectures, the number of parameters, and the root mean squared error in cm obtained by our solution on the MPIIFaceGaze [5] dataset. We achieve state-of-the-art performance, obtaining *d* = 3.64 cm, significantly improving over the *d* = 3.89 cm obtained by the EFE method proposed by [6]. Our approach reaches this remarkable performance while having notably fewer parameters compared to the second-best approach from the literature: 0.373M vs. 22M. Many of the previously published works in the literature use computationally expensive backbones that are not optimized for our task of choice. Therefore, we see methods employing backbones having millions of parameters without a clear requirement for this. In this regard, we opt for a straightforward method that has been designed to satisfy the minimum requirements for performing PoG estimation, and not overshoot in terms of resources used. This clearly displays the unnecessary use of models that are too complex for solving this task, and shows how we seized the opportunity to come up with a more suitable architecture for this particular problem, further underlining our contribution.

### 5.2. Benchmarking Our Dataset

One important point to mention is that due to the cross-person and cross-device variation, our calibration-free data collection approach inevitably has a small acquisition noise of less than 50 pixels. Nevertheless, all methods from the literature suffer from this type of shortcoming, as no RGB camera-based data gathering approach proved to be perfect.

With that out of the way, once more the 15-fold cross-validation methodology was adopted so that the reported results were consistent with the benchmarks performed on the other datasets from the literature.

Moreover, the extensive ablation study performed on our dataset is displayed in Table 4. The purpose of reporting the parameters is solely because we compare our baseline model with an ensemble variation (see second-to-last row).

While the first row displays the bare-bones training of our model, for setting the simplest possible baseline, the second row displays the possibility of pre-training our model on the MPIIFaceGaze dataset [8], so that through transfer learning we can evaluate PoG estimation on our dataset. Thus, it can be noticed that models can generalize well on our data.

The next three lines aim to evaluate how our model behaves when pre-training or augmentations are applied, and monitor its improvement. Interestingly enough, the model performs best when pre-trained, and then, fine-tuned on our data using augmentations.

Afterward, we test what happens when we consider an ensemble composed of two models of the same kind that have been trained according to the previously best approach. Our methodology is rather basic, and it consists of averaging the output of the two individually trained models, which determines the overall ensemble prediction.

Lastly, employing a state-of-the-art face detection model provides us with the bounding boxes of the subjects from our collected data, then the given crops are used to train and evaluate our model. In this scenario, the performance improvement is significant. Yet, this trade-off makes the inference pipeline incompatible with real-time performance, leading to the effective defeat of the purpose of our contribution.

As observed, a couple of better approaches have been found in this ablation study at the expense of having additional pre-processed information, whereas our best approach (trained on own data with pre-training and augmentations) that uses the whole-image RGB input achieves remarkable results without sacrificing time and space efficiency. On the one hand, the ensemble approach proves to bring a significant improvement. On the other hand, the increase provided by doubling the parameter count may not be enough to sacrifice the efficiency of our best model so far. Nonetheless, the best approach is the one that has additional annotations, but considering the increasing need for data, the labeling effort quickly becomes overwhelming in the context of deep learning. So, our automatically annotated dataset and best model provide the ultimate bundle performance-wise while being resource-effective as well.

## 6. Discussion

Considering the context of our task of choice, PoG estimation proved to be a challenging task, both when considering the data acquisition process and the model performance. Firstly, every acquisition pipeline has its inherent errors and limitations. Nonetheless, we managed to reduce the risks of error occurrences while maximizing the data acquisition rate. Compared to other existing methodologies [1,7], ours is boiled down to the sole purpose of acquiring PoG estimation data easily. Secondly, the compared works tackle multiple tasks that have a higher complexity than PoG estimation, or their input data consist of more annotated elements. When designing the proposed solution only for our task of choice, the resulting architecture was crafted with efficiency in mind, while the previously existing solutions [5] were not specific enough to benefit from the same advantages as our model.

Future improvements may concern collecting more data using crowdsourcing platforms, using hardware-specific operations to further improve the runtime performance of deployed embedded models, and using noise injection in the input images to obtain a more robust architecture.

Incorporating a more diverse set of subjects in future studies would also be valuable for assessing the model’s robustness and its ability to generalize across various demographic groups. Differences in gaze behavior and visual patterns among different age groups, genders, or ethnicities could affect the model’s accuracy and reliability. A broader demographic representation would help ensure that the model performs effectively and equitably across different user populations. Addressing these aspects will not only improve the model’s generalization but also contribute to the development of more inclusive and representative gaze tracking systems.

## 7. Conclusions

In conclusion, we have reviewed the current PoG estimation datasets from the literature, identified their weak points, and introduced a simpler methodology that helped us gather automatically and reliably annotated data in varying everyday conditions. Furthermore, we proposed an efficient CNN approach for our task of interest that was specifically tailored for real-time performance. Then, not only did we discuss the motivation for our choice of architecture, train the model on one of the most relevant PoG estimation datasets, and prove that it successfully achieved SotA performance while having fewer parameters compared to existing approaches, but we also performed an ablation study on our novel dataset and confirmed that our experimental setup is sound and able to perform well without the need for camera calibration.

However, it is essential to address the ethical and social implications of implementing PoG technologies, particularly concerning data privacy and security. PoG systems inherently involve the collection and analysis of sensitive information about users’ gaze patterns. This raises several critical issues:

- Data privacy: Ensuring the confidentiality and protection of users’ gaze data is paramount. Since gaze patterns can reveal personal information and behavioral tendencies, robust measures must be in place to prevent unauthorized access and misuse of this data. Effective anonymization techniques and strict data access controls are necessary to safeguard user privacy.
- Data security: The security of the data collected by PoG systems needs to be ensured through encryption and secure storage solutions. As PoG technologies become more integrated into various applications, the potential for data breaches increases, making it crucial to implement comprehensive security protocols to protect against cyber threats.
- Ethical use: Clear guidelines and regulations should be established regarding the ethical use of gaze data. This includes transparency about how the data will be used, ensuring that they are not employed for purposes beyond the scope of user consent, and addressing any potential biases that may arise in the data collection and analysis processes.

## Figures and Tables

**Table 1:** Proposed CNN architecture. BN = batch normalization; C = convolution; D = dropout; R = ReLU; GAPooling = global average pooling.

Block	Layer	K	#Params	Dimensions
0	Input image	-	-	H × W × 3
1	C+R+D(0,3)	3 × 3	1792	H − 2 × W − 2 × 64
1	Maxpooling	2 × 2	-	(H − 2)/2 × (W− 2)/2 × 64
2	C+R+D(0,3)	3 × 3	36,928	(H − 2)/2 × (W − 2)/2 × 64
2	BN	-	256	(H − 2)/2 × (W − 2)/2 × 64
3	C+R+D(0,3)	3 × 3	36,928	(H− 2)/2 × (W−2)/2 × 64
3	BN	-	256	(H − 2)/2 × (W− 2)/2 × 64
4	C+R+D(0,3)	3 × 3	36,928	(H − 2)/2 × (W − 2)/2 × 64
4	BN	-	256	(H − 2)/2 × (W − 2)/2 × 64
5	C+R+D(0,3)	3 × 3	36,928	(H − 2)/2−2 × (W − 2)/2 − 2 × 64
5	BN	-	256	(H−2)/2−2 × (W − 2)/2 − 2 × 64
6	C+R+D(0,3)	3 × 3	36,928	(H − 2)/2−4 × (W − 2)/2−4×64
6	BN	-	256	(H−2)/2−4 × (W − 2)/2 − 4 × 64
7	C+R+D(0,3)	3 × 3	36,928	(H − 2)/2−4 × (W − 2)/2 − 4 × 64
7	BN	-	256	(H−2)/2−4 × (W − 2)/2 − 4 × 64
8	C+R+D(0,3)	3 × 3	36,928	(H − 2)/2 − 4 × (W − 2)/2 − 4 × 64
8	BN	-	256	(H − 2)/2 − 4 × (W − 2)/2 − 4 × 64
9	C+R+D(0,3)	3 × 3	36,928	(H − 2)/2 − 6 × (W − 2)/2 − 6 × 64
9	BN	-	256	(H − 2)/2 − 6 × (W − 2)/2 − 6 × 64
10	C+R+D(0,3)	3 × 3	36,928	(H − 2)/2 − 8 × (W − 2)/2 − 8 × 64
10	BN	-	256	(H − 2)/2 − 8 × (W − 2)/2 − 8 × 64
11	C+R+D(0,3)	3 × 3	36,928	(H − 2)/2 − 10 × (W − 2)/2−10 × 64
11	BN	-	256	(H − 2)/2 − 10 × (W − 2)/2 − 10 × 64
12	GAPooling			64
12	D(0.3)	-	-	64
13	Dense(2)		130	2
-	Output		373,762	

**Table 2:** Overview of the collected data: screen resolution (W—width; H—height) is provided both in pixels (px) and millimeters (mm). The second to last column mentions the webcam resolution in pixels, and the last column counts the collected samples per subject.

#ID	W (px)	H (px)	W (mm)	H (mm)	Distance (cm)	Webcam Resolution	#Images
1	1920	1080	344.4	193.7	60–80	640 × 480	600
2	2560	1440	610	340	60–80	1920 × 1080	526
3	1920	1080	312	177	45–70	640 × 480	1009
4	1920	1080	380	215	45–65	1920 × 1080	626
5	2256	1504	297	229	50–60	1920 × 1080	1106
6	1920	1080	345	196	30–45	640 × 480	778
7	1920	1080	344	194	95–105	1280 × 720	500
8	1920	1080	345.3	194.2	45–55	640 × 480	478
9	2560	1600	350	200	40–50	640× 480	600
10	2560	1600	677.33	423.33	50–60	640 × 480	614
11	1920	1080	362	258	45–55	1280 × 720	535
12	1280	720	338.66	161.88	30–45	1280 × 720	643
13	1920	1080	344	196	55–65	1280 × 720	600
14	2880	1800	310	200	40–65	1280 × 720	600
15	1920	1080	382	215	40–55	1280 × 720	600
16	1920	1080	344	193	45–60	640 × 480	816
17	1920	1080	344	194	30–60	640 × 480	913
18	1920	1080	344	194	40–60	640 × 480	685
19	1920	1080	344.4	193.7	40–60	640 × 480	700
20	1920	1080	344	193	40–50	640 ×480	600
21	1920	1080	343.5	193.2	40–50	640 × 480	500
22	1728	1117	344.2	222.5	50–60	1920 ×1080	501
23	1920	1080	344.4	193.7	60–80	640 × 480	369
24	1920	1080	530.3	298.3	55–70	640 × 480	492
25	1920	1080	343.5	193.2	65-80	640 × 480	344
26	1920	1080	344.4	193.7	50–65	640 × 480	526
27	1366	768	343.6	193.2	40–60	640 × 480	682
28	1920	1080	350	200	35–45	640 × 480	599
29	1920	1200	344	193	50–60	1280 × 720	600
30	1728	1117	344.2	222.5	60–75	1920 × 1080	404
31	2880	1800	302.1	188.8	50–65	640 × 480	535
32	1920	1080	344.4	193.7	50–65	640 × 480	634
33	2496	1664	317	212	45–60	640×480	506
**ALL**							**20221**

**Table 3:** Our approach compared to current state-of-the-art approaches on the MPIIFaceGaze dataset [5].

Paper	Backbone	# Params	Error (cm)
Ours	Ours	0.373M	3.64
EFE [6]	EfficientNetV2 S [13]	22M	3.89
Appearance-Based [5]	Spatial Weights CNN [5]	196M	4.21
MPIIFaceGaze [5]	AlexNet [14]	53M	4.61

**Table 4:** Ablation study performed on our novel dataset.

Own Data Used	Pretrain	Augmentation	# Params	Error (cm)
yes	-	-	373,762	12.03
no	yes	-	373.762	11.62
yes	no	yes	373,762	11.4
yes	yes	-	373,762	7.37
yes	yes	yes	373,762	6.02
yes	yes	yes	2 × 373,762	5.64
yes + face detected	yes	yes	373,762	3.98

## Data Availability

The dataset is restricted due to privacy and ethical considerations. Researchers interested in accessing the data should contact the corresponding author for further information and potential access. Any data sharing will be subject to approval and may require adherence to specific ethical guidelines and confidentiality agreements.

## Abbreviations

The following abbreviations are used in this manuscript:

PoG	Point of gaze
IR	Infrared
CNN	Convolutional neural network
SotA	State of the art
ReLU	Rectified linear unit
BN	Batch normalization
W	Width
H	Height
px	Pixels
mm	Millimeters
cm	Centimeters
